# Satellite microglia: marker of traumatic brain injury and regulator of neuronal excitability

**DOI:** 10.1186/s12974-024-03328-9

**Published:** 2025-01-16

**Authors:** Alicia B. Feichtenbiner, Karinn Sytsma, Ryan P. O’Boyle, Rhonda Mittenzwei, Heather Maioli, Kathryn P. Scherpelz, Daniel D. Child, Ning Li, Jeanelle Ariza Torres, Lisa Keene, Amanda Kirkland, Kimberly Howard, Caitlin Latimer, C. Dirk Keene, Christopher Ransom, Amber L. Nolan

**Affiliations:** 1 Department of Laboratory Medicine and Pathology, University of Washington, Seattle, WA 98104 USA; 2 Miller School of Medicine, University of Miami, Miami, FL 33136 USA; 3 King County Office of the Medical Examiner, Seattle, WA 98104 USA; 4 Office of Chief Medical Examiner of the City of New York, New York, NY 10016 USA; 5 Department of Neurology, University of Washington, Seattle, WA 98195 USA; 6 Puget Sound Veterans Affairs Seattle Medical Center, Seattle, WA 98108 USA; 7 Allen Institute, Seattle, WA 98109 USA

**Keywords:** Neuropathology, Traumatic brain injury, Microglia, Cortical circuits, Electrophysiology

## Abstract

Traumatic brain injury is a leading cause of chronic neurologic disability and a risk factor for development of neurodegenerative disease. However, little is known regarding the pathophysiology of human traumatic brain injury, especially in the window after acute injury and the later life development of progressive neurodegenerative disease. Given the proposed mechanisms of toxic protein production and neuroinflammation as possible initiators or contributors to progressive pathology, we examined phosphorylated tau accumulation, microgliosis and astrogliosis using immunostaining in the orbitofrontal cortex, a region often vulnerable across traumatic brain injury exposures, in an age and sex-matched cohort of community traumatic brain injury including both mild and severe cases in midlife. We found that microglial response is most prominent after chronic traumatic brain injury, and interactions with neurons in the form of satellite microglia are increased, even after mild traumatic brain injury. Taking our investigation into a mouse model, we identified that these satellite microglia suppress neuronal excitability in control conditions but lose this ability with chronic traumatic brain injury. At the same time, network hyperexcitability is present in both mouse and human orbitofrontal cortex. Our findings support a role for loss of homeostatic control by satellite microglia in the maladaptive circuit changes that occur after traumatic brain injury.

**Supplementary Information:**

The online version contains supplementary material available at 10.1186/s12974-024-03328-9.

## Introduction

Traumatic brain injury (TBI) is a risk factor for dementia and neurodegenerative disease (NDD) [2–4], but the mechanisms occurring between early life TBI and later NDD development are unknown. Furthermore, only few studies have examined the human neuropathology of TBI at younger ages before NDD has fully developed [5, 6], limiting our knowledge. Toxic protein production is proposed to result after TBI, progressively accumulate and spread through a prion-like mechanism [7, 8]. Tau is one such protein that contributes to microtubule function and axonal transport [9] that can be disassociated from axons with injury [7]. Accumulation of abnormal tau is a hallmark of chronic traumatic encephalopathy and Alzheimer’s disease, the most common NDDs associated with TBI [10, 11]. “Neuroinflammation” or reactive gliosis also occurs alongside injury and may contribute to progressive pathology after TBI [12], with astrocytes and microglia thought to produce inflammatory cytokines, inappropriately phagocytize synapses and/or contribute to cell death [13–17]. However, few human tissue studies have carefully looked at the timeframe after acute injury but before the later onset of dementia to see if abnormal protein accumulation and/or neuroinflammation continues and the details of cellular interactions.

There is also intense debate about the involvement of chronic microglial activation in neurological disease progression [18]. Both depletion and repopulation of microglia can improve function in many pre-clinical models of neurologic disease [19–24], supporting a direct contribution to progressive pathology. Microglia also have an increasingly recognized role in regulating neuronal activity [25–29], and it is not understood if or how microglia’s impact on neuronal excitability contributes or protects in disease. One microglial subtype that may be critical in the regulation of neuronal excitability is the perineuronal satellite microglia (Sat-MG). These microglia are juxtaposed adjacent to neurons and entwine their soma and processes around the neuronal cell body [30, 31], often interacting with the region of axon potential production [32]. Sat-MG were described more than 100 years ago by Río-Hortega in his original description of microglia [31], yet little attention has been paid to understanding the functional properties of this subtype, especially in the context of modifying neuronal activity. Recent studies find that Sat-MG display spontaneous electrical activity with different characteristics when compared to non-satellite microglia, supporting a unique function for this subtype [33]. Furthermore, these Sat-MG do not extend or retract processes like other microglia, but instead have filipodia that make frequent contact with the neuronal surface, again suggesting a different function [34]. Whether this subtype of microglia contributes to the chronic neuroinflammatory response in human TBI has not been evaluated nor its contribution to neuronal function at baseline and after chronic injury.

Thus, we evaluated human neuropathology at chronic time points after TBI, focusing on accumulation of tau and neuroinflammatory changes. We find that microglial response is most prominent after chronic TBI, and interactions with neurons in the form of satellite microglia are increased, even after mild TBI. We take these findings into a mouse model of contusion to further examine how satellite microglia are involved with neuronal function before and after injury. Our results provide mechanistic insight into how satellite microglia modulate neuronal activity at chronic time points following TBI.

## Methods

### Human tissues

All studies at the University of Washington (UW) utilizing brain donation are approved by the UW School of Medicine Compliance office and Institutional Review Board and collected with informed consent. Brain donors in this study were part of the Pacific Northwest Brain Donor Network, which collects tissue from military veterans and civilians, both with and without a history of traumatic brain injury in collaboration with local Seattle area medical examiners. Consent for brain donation was obtained from the legal next of kin who agreed to be contacted and were interested in the UW research program. History of TBI (“Did they ever have a head injury?”) and other metrics were obtained via family questionnaire. Data collected included mechanisms of TBI, loss of consciousness, symptoms after the injury, and medical and psychiatric conditions. Control cases never had a known head injury or other history of neurologic disease. Mild TBI cases were defined as head injury with associated symptoms after the injury such as headaches, sleep disturbances, slower processing, memory loss/etc. Severe TBI cases were defined as the presence of a head injury associated with coma/hospitalization greater than 1 week or skull fractures/contusions. It should be noted that head injuries recognized by these donor families were predominately isolated TBI events except for one mild TBI donor with a notable 12-year boxing history and continuous head injuries over that span; 40% of TBI cases also had multiple events isolated in time and data in Table 1 indicate the most severe or most recent injury from the families’ perspective. In addition, several cases across control and TBI groups had a contact sport and/or military service history and additional exposure to “mild repetitive TBI” that the family did not consider head injury is possible. Cases were excluded for neurologic history before head injury or separate neurologic injury after the TBI, such as stroke. Ten cases per group were selected that could be age- and sex- matched (see Table 1).

**Table 1:** Demographics, TBI characteristics and pathology of the human tissue donors

	Controls	Mild TBI (mT)	Severe TBI (sT)
Demographics			
Number	10	10	10
Age (SD)	49.6 (10.0)	49.1 (10.3)	49.2 (11.8)
% Male	100	100	100
% Military service history	50	70	20
% Contact sports history	20	60	30
TBI data			
Years post injury	–	14.4 (12.9)	19.4 (15.7)
% MVA	–	40%	60%
% Fall from height	–	10%	30%
% Falling object	–	20%	–
% Explosion/blast	–	20%	10%
% Sport-related	–	30%	20%
% Repeated TBIs	–	40%	40%
% LOC	–	70%	100%
% Post-TBI seizures	–	30%	60%
Pathology			
% Contusion (in OFC)	0%	0%	70% (40%)
% + CTE	0%	40%	30%
% ≥ ADRC Intermediate	0%	0%	0%
% + LBD	0%	10%	10%
% + TDP-43	0%	10%	10%

Every case underwent a neuropathologic evaluation that included full assessment for neurodegenerative disease by current consensus criteria including Alzheimer’s disease, Lewy body disease, TDP-43 pathologies, chronic traumatic encephalopathy (CTE), and other tauopathies as described in Latimer et al. [35]. Over 17 cortical regions with numerous sulci sampled were stained with phosphorylated tau (AT8) to evaluate for CTE pathology. Bilateral orbitofrontal and anterior temporal lobes were also always evaluated to assess for contusion in every case. For this study, the OFC was chosen for microscopic evaluation given its frequent vulnerability in mild to severe TBI [36, 37].

### Mice

All experiments were conducted in accordance with the National Institutes of Health Guide for the Care and Use of Laboratory Animals and approved by the Institutional Animal Care and Use Committee of the University of Washington and the Puget Sound Veterans Affairs Medical Center. Male and female mice were bred in-house using the strain: C57BL/6-Tmem119em2Gfng/J (Tmem119, catalog #031823, The Jackson Laboratory, RRID: IMSR_JAX:031823). Mice were group housed in environmentally controlled conditions with a 12 h light/dark cycle at 21 ± 1 °C, ∼50% humidity, and provided with food and water ad libitum. Mice were 11–13 weeks of age at the time of surgery. Both males and females were used, and results from both sexes were pooled. We did not observe any obvious influence or association of sex on the experimental outcomes.

### Surgical procedures

Each cage of mice (2–5 animals) was split to randomly assign half to the TBI or sham surgery group. The mice were anesthetized with 4% isoflurane for 2–5 min, weighed, and the surgical area was shaved. Mice were secured within a stereotaxic frame and isoflurane anesthesia was decreased to 2%. A frontal lobe contusion, a moderate-severe traumatic brain injury, was performed as previously described, with parameters chosen to induce a contusion injury in the right frontal lobe without resulting in a large cavity [1, 38]. An incision along the midline of the skull was used to visualize bregma and the injury location. A ~ 2.5 mm craniectomy was performed at + 2.34 mm anteroposterior and + 1.63 mm mediolateral with respect to bregma. A 2 mm convex tip attached to an electromagnetic impactor (Leica) was used to induce a 1.25 mm deep controlled cortical impact with a hit at a velocity of 4.0 m/s sustained for 300 ms. The sham procedure followed the same protocol, substituting a light touch of the drill to the skull for the craniectomy and impact. In some cases, a full craniectomy was also performed without impact for comparison (see Supplementary material). Following the procedure, the incision was closed with suture, and the mice were moved to a recovery cage, warmed with a heating pad. All mice were returned to their original cages once they were awake and alert.

### Slice preparation

Sagittal brain slices were prepared at a thickness of 250 μm from mice at ~ 1 week and ~ 2 months following the TBI or sham procedure or in mice ~ 3–5 months of age without a surgical procedure. Mice were anesthetized with 4–5% isoflurane for 3–5 min prior to decapitation. The brain was isolated and placed in an ice-cold sucrose solution containing (in mM): 210 sucrose, 1.25 NaH_2_PO_4_, 25 NaHCO_3_, 2.5 KCl, 0.5 CaCl_2_, 7 MgCl_2_, 7 dextrose, 1.3 ascorbic acid, 3 sodium pyruvate (bubbled with 95% O_2_/5% CO_2_, pH ~ 7.4). The brain was cut on a slice vibratome (VT1200, Leica Biosystems). Slices were maintained in a holding solution containing (in mM): 125 NaCl, 2.5 KCl, 1.25 NaH_2_PO_4_, 25 NaHCO_3_. 2 CaCl_2_, 2 MgCl_2_, 10 dextrose, 1.3 ascorbic acid, 3 sodium pyruvate (bubbled with 95% O_2_/5% CO_2_, pH ~ 7.4 at 37 °C for 30 min and then room temperature until recording). To test the effects of blocking the microglial-specific P2Y12 receptor, the antagonist PSB 0739 1 μM [39] was included in the holding solution and subsequent recording solution. Slices were incubated with the antagonist for at least one hour prior to recording.

### Intracellular recording

Whole-cell patch clamp recordings were obtained from slices in a submersion chamber heated to 30–34 °C containing an artificial CSF (aCSF) solution with the following concentrations (in mM): 125 NaCl, 3 KCl, 1.25 NaH_2_PO_4_, 25 HCO_3_, 2 CaCl_2_, 1 MgCl_2_, 10 dextrose (bubbled with 95% O_2_/5% CO_2_, pH ~ 7.4). Patch pipettes (2-5 MΩ) were pulled from filamented borosilicate glass capillaries (catalog #BF100-58–10, Sutter Instruments). An intracellular solution containing (in mM): 135 K-Gluconate, 10 HEPES, 4 NaCl, 1 EGTA, 4 MgATP, 0.5 Na_3_GTP, and in some cases 1–2% biocytin, was used to fill the patch pipettes.

Under a 10 × objective, the OFC cortex was located and the objective was centered over layer V. Neurons, specifically pyramidal neurons, were identified with a 40 × water-immersion objective using infrared microscopy (Scientifica). Fluorescence was then used to identify microglia expressing GFP and target neurons away or next to a satellite microglia. Recordings were made using a MultiClamp700B amplifier, which was connected to the computer with a Digidata 550B ADC and recorded at a sampling rate of 20 kHz with pClamp software (Molecular Devices). We did not correct for the junction potential, but pipette capacitance was appropriately compensated. Cells were excluded if the access resistance was or rose above 20 MΩ during recording.

The passive membrane and active action potential (AP) spiking characteristics were assessed by injection of a series of hyperpolarizing and depolarizing current steps with a duration of 250 ms from − 250 to 700 pA (50 pA increments). The resting membrane potential was the measured voltage of the cell after obtaining whole-cell configuration. A holding current was then applied to maintain the neuron at ~ − 65 mV before/after current injections. The input resistance was determined from the steady-state voltage reached during the − 50 pA current injection. To ensure recording from a similarly sized neuronal population, neurons with an input resistance > 200 MOhms were excluded.

AP properties including the half width, threshold, amplitude, rising slope, falling slope, and spike afterhyperpolarization (AHP) were calculated based on the response to a current pulse that was 100 pA above the minimal level that elicited spiking, or if this injection had fewer than three action potentials, the first current injection with > 3 action potentials. The AP threshold was defined as the voltage at which the third derivative of V (d^3^V/dt) was maximal just before the action potential peak. The AP amplitude was calculated by measuring the voltage difference between the peak voltage of the action potential and the spike threshold. The half width was determined as the duration of the action potential at half the amplitude. The spike AHP was the voltage difference between the AP threshold and the minimum voltage before the next AP. The rising and falling slopes of the AP were the maximum of the first derivative of V between the threshold to the peak amplitude and the peak amplitude to the minimum voltage before the next AP, respectively.

Action potential timing was detected by recording the time at which the positive slope of the membrane potential crossed 0 mV. From the action potential times, the instantaneous frequency for each action potential was determined (1/interspike interval). The adaptation index of each cell was the ratio of the first over the last instantaneous firing frequency in response to a current pulse. The AP rate as a function of current injection was examined by plotting the first instantaneous AP frequency versus current injection amplitude.

### Network excitability

To evaluate network excitability in vitro, slices were incubated in aCSF without Mg^2+^ (0 mM MgCl_2_) and with increased K^+^ (5 mM KCl). In this solution, synchronized polysynaptic events occur with time, and the time to onset of the second clearly recurrent polysynaptic burst with an amplitude > 2 mV was compared while recording in current clamp from layer V neurons while holding the membrane potential to ~ − 65 mV with holding current [38]. The antagonist PSB 0739 5 μM [39] was also included to test the effect of P2Y12R antagonism in some experiments. Data were analyzed blinded to condition.

### Immunohistochemistry/fluorescent staining

For immunofluorescent staining, 20 µm human brain sections fixed in paraffin were deparaffinized via 5 min rinses in each: xylenes × 3, 100% isopropanol × 2, 96% isopropanol, 70% isopropanol, 50% isopropanol, then rinsed in TBST (1X Tris Buffered Saline with 0.5% Triton X-100). Tissue was then decloaked in a Diva solution (50–828-31, Biocare Medical) for 8 min at 110 ºC, rinsed in TBST, then decloaked again in Nuclear Decloaker solution (50–823-96, Biocare Medical) for 8 min at 110 ºC, and rinsed in TBST. Slides were washed in 10% beta-cyclodextrine solution at 65 ºC with constant motion overnight, refreshing solution hourly. Tissue was incubated for 1 h in blocking solution (10% donkey solution diluted in TBST), then overnight with primary antibodies, IBA-1 (a marker of microglia/macrophages, 1:500, 019–19741, Wako) with or without Cfos (a marker of neuronal activation, 1:50, LS-C93966, LSBio), diluted in blocking solution. The following day, slides were rinsed in TBST, then incubated for 2 h with secondary antibodies (1:500, Alexa 488 donkey anti-rabbit, A-21206, Invitrogen; and 1:500, Alexa 647 donkey anti-sheep, A-21448, Invitrogen) and Nissl (1:500, NeuroTrace 530/615, N21482, Invitrogen). Slides were rinsed and mounted in an aqueous medium. For control, a second set of slides was stained omitting the primary antibodies but including all other reagents. The control slides with only secondary antibodies were analyzed to ensure all staining was brighter than any remaining autofluorescence and imaging parameters were adjusted accordingly.

Mouse brain slices obtained from electrophysiological recordings were drop-fixed in 4% paraformaldehyde in a phosphate-buffered solution (4–48 h), then rinsed with phosphate-buffered saline (PBS) (0.1 M PBS) and stored at 4 °C for 0–5 days until processing. These slices were stained as floating sections. Mouse brain cryosections were also obtained after perfusion at 40 μm. Mice were perfused with formalin and the brain removed. Mouse brains sat for 24 h in formalin, followed by 24 h in 15% sucrose, and then stored in 30% sucrose. Mouse brains were frozen in an OCT/30% sucrose mixture (2:1 ratio) and kept at – 80 ºC. Slices were cut at 40 μm using a cryostat (CM1950, Leica Biosystems).

For both floating and cryo-sections, sections were washed in PBS (3 × 10 min), incubated in a blocking solution (10% donkey serum diluted in 0.5% Triton X-100 in 0.1 M PBS) for 1 h, and then incubated with primary antibody, anti-GFP (expressed in microglia under the Tmem119 promoter, 1:500, 11–476-C100, Exbio) with 0.25% Triton X-100, and 5% donkey serum in 0.1 M PBS, at room temperature overnight, along with streptavidin-conjugated Alexa-555 (1:500, S32355, ThermoFisher Scientific) or Nissl (1:100, NeuroTrace 530/615, N21482, Invitrogen) and in some cases anti-Cfos (1:50, ab208942, Abcam). The following day, sections were then rinsed with PBS (3 × 10 min) and incubated in secondary antibody (1:500, Alexa 488 donkey anti-rabbit, Invitrogen, A21206; 1:500, Alexa 647 donkey anti-mouse, A-31571, Thermofisher) at room temperature for 4 h. Finally, sections were rinsed and mounted in an aqueous medium.

To obtain images of immunofluorescent staining in mouse and human tissues, 10 µM Z-stack images were taken using a Leica SP8X Confocal at 40 × or 63 × magnification. In mice, images were used to count the number of neurons with satellite microglia, identify the percentage of neurons close to and away from microglia with positive cfos expression or confirm the relationship of satellite microglia to recorded neurons. In human tissues, images were used to quantify the number of neurons with satellite microglia, perform 3D analysis in Imaris as described below, and identify the percentage of neurons close to and away from microglia with positive cfos expression. All image analysis was performed blinded to condition.

For DAB (3,3′-Diaminobenzidine) immunohistochemical staining of human tissues, 20 µM sections of the OFC were immunostained by a BioCare intelliPATH^™^ autostainer with AT8 (a phosphorylated tau antibody, 1:800, MN1020, Thermofisher), IBA-1 (a microglia/macrophage marker, 1:1000, 019–19741, Wako), CD68 (a marker of lysosomes, increased in reactive microglia, 1:500, M0814, DAKO) or GFAP (a marker of reactive astrocytes, 1:1000, Z033401-2, DAKO) antibodies, appropriate horseradish peroxidase (HRP)-conjugated secondary antibodies and then counterstained with hematoxylin for evaluation (see [35] for more detailed protocols). Slides were scanned with an Aperio AT2 Leica slide scanner at 20x. Images were opened in Halo software to perform analysis described below blinded to condition.

### Image analysis

To determine if a neuron was adjacent to a satellite microglia, z-stack images were opened in Image J. To count as a satellite microglia, the soma of the microglia had to be in contact with the neuronal soma, as well as the microglial processes touching or wrapping around the neuronal soma. Images were analyzed in 3D across z-stacks to ensure microglia were in the same plane. To determine cfos-positivity, images were taken with the same parameters on the confocal microscope and opened in imageJ using the same minimum and maximum thresholds. A cell was determined to be positive if there was increased staining in the soma (seen with Nissl stain) above the background. For each image, the total number of neurons, neurons adjacent to satellite microglia, cfos-positive neurons, and cfos-positive neurons adjacent to satellite microglia were manually counted using the ImageJ Cell Counter.

### Imaris analyis

LIF files were loaded into Imaris 10.0.0 for analysis. Using the surfaces tool, a mask was drawn to isolate the microglia or neuron in the 63 × image. The surfaces tool was then used again on the masked image, with the smooth factor set to 0.1 μm. For microglia, background subtraction with the largest sphere diameter used for thresholding was set to 0.676 μm, and the active threshold was set to the point at which the surface was shown to cover the entirety of the microglia or neuron until only a faint outline of the fluorescence was visible in the image. For neurons, LabKit for Pixel Classification was used. Pixels were determined to be foreground or background by researcher (ABF) until the automated LabKit segmentation created a solid neuron with minimal background. Surface volume and microglia-neuron volume overlap was provided by Imaris with object-object statistics. Contact surface area was determined using the Surface-Surface Contact Area XT (Matthew Gastinger, imaris.oxinst.com). The filaments tool was used with the tree algorithm autopath (no loops and no spine). The estimated largest diameter of the starting point was determined by adjusting the point diameter to the minimal size in which the box contained the entirety of the soma. The starting points threshold was adjusted to one, with the only remaining starting point lying on the soma of the microglia. Using the soma model, the seed points thinnest diameter was set to the minimal size in which the box contained the smallest section of any of the processes. Seed points threshold was adjusted until seed points populated the majority of the microglia and processes. The AI filament model was then trained by selecting seed points on the processes to keep and those in the background to discard and retrained by adjusting these selections until the kept seed points fell only on the processes and everything else was discarded. Segment classification was conducted in the same manner as seed point classification. Filament lengths less than 0.9 μm were discarded. Filament no. sholl intersections was provided by Imaris with a spheres resolution of 1 μm. The soma of the microglia was isolated using a free-handed surface to mask the rest of the image, and then absolute intensity thresholding was used to create the surface, with the active threshold value set using the same process as that with the entire microglia and background subtraction. Volume was recorded and the soma surface was imported into the filament tool as the proper soma.

### Halo analysis

SVS images were loaded into HALO image analysis software (Indica Labs) for 2-dimensional image analysis. For every image, grey matter was annotated and the Area Quantification module was used to quantify the percent area of staining in the grey matter. The area quantification modules were created by color picking the stain from an image and adjusting the minimum optical density of the stain to be considered positive while also avoiding background stain. Developed analysis settings were checked with multiple images for each stain, and batch analyzed in annotated areas using the same parameters across images.

### Statistical analysis

All data were evaluated with GraphPad Prism 9 statistical software. For the various electrophysiological analyses, we repeated the experiment in multiple cohorts of animals. Statistical significance between groups for human tissue staining/analyses was determined with either a repeated measures one-way ANOVA, mixed effects analysis, paired *t* test, or Wilcoxon test (for nonparametric variables). For mouse experiments, statistical significance between groups for most variables was determined using an unpaired, two-tailed *t* test with or without Welch’s correction. For nonparametric data, a two-tailed Mann–Whitney test was assessed to determine significance. The firing responses to increasing current injections were analyzed as a repeated measures two-way ANOVA. Cfos staining was analyzed with a two-way ANOVA in both mouse and human tissues. Post hoc multiple comparisons were assessed controlling for the false discovery rate using the method of Benjamini and Hochberg. *p* values < 0.05 were considered significant. All the statistical details of experiments can be found in the figure legends including the statistical tests used, exact value of *n*, and what *n* represents.

## Results

### Cohort demographics and neuropathological assessment

Table 1 summarizes the clinical characteristics of the 30 cases included in this study. All cases available for matching across groups by sex and age were male with an average age around 49 years, spanning from 25 to 62. TBI exposures occurred due to a range of mechanisms, from falls and motor vehicle accidents to sports-related injuries, most occurring years before death. Military service and contact sports histories were common with the highest levels noted in the mild TBI (mT) groups. Significant Alzheimer’s disease neuropathologic change was not found in any case, but brainstem Lewy body disease (LBD) was present in one 48 year-old severe TBI (sT) case and limbic stage LBD was present in one 57-year-old mT case. TDP-43 pathology limited to limbic-predominant age-related TDP-43 encephalopathy (LATE) stage 1 was present in two TBI cases. CTE pathology was more frequent, but only found in donors with TBI history (40% in mT and 30% in sT). Chronic brain contusion was also noted in 70% of sT cases, 40% of which were in the analyzed OFC region.

### Microglial response is most prominent in the human orbitofrontal cortex after TBI

**Fig. 1:**
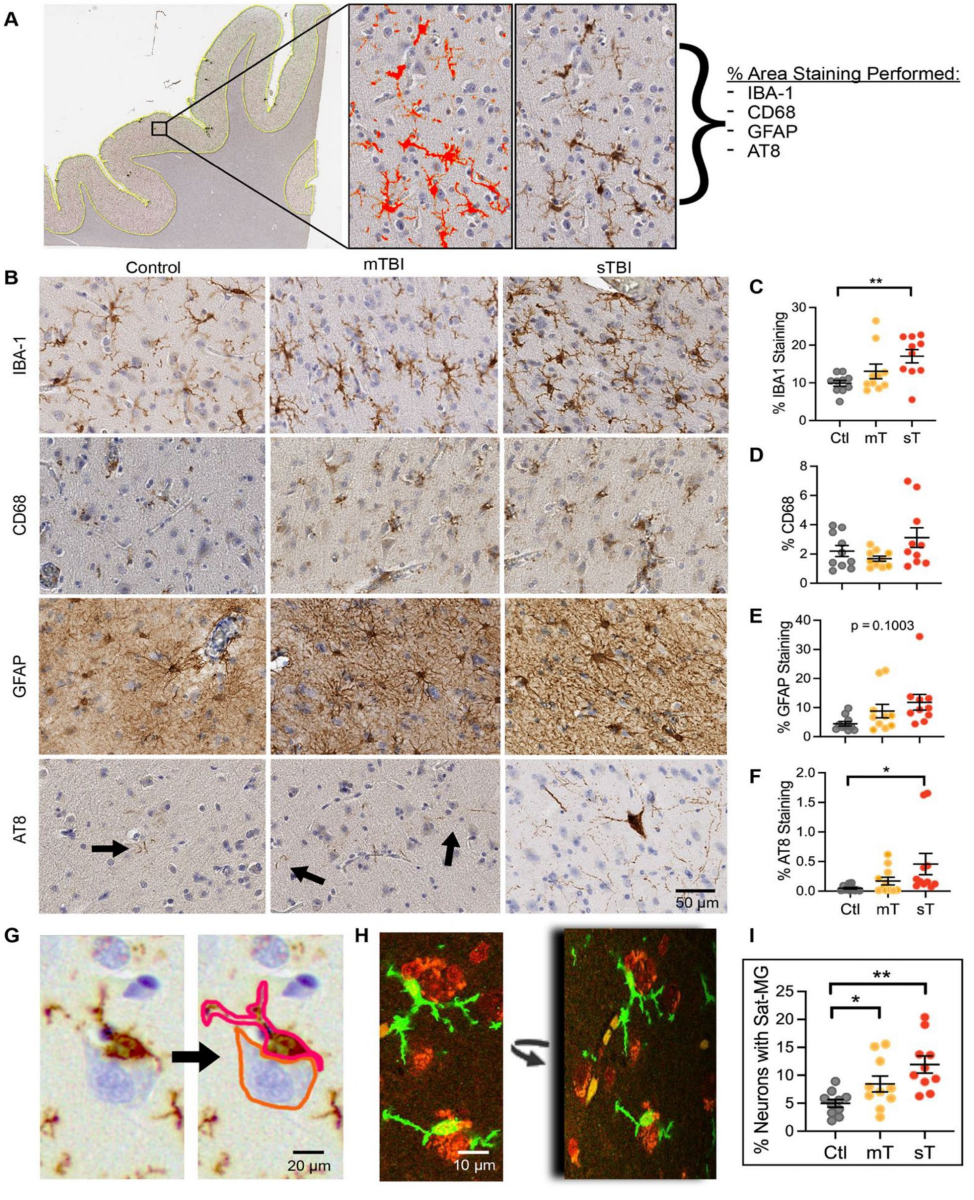
Prominent changes in microglia, including satellite microglia, in the human orbitofrontal cortex after chronic TBI. **A** Representative image demonstrating the Halo image analysis protocol to determine percentage area of staining. Annotation of the grey matter is denoted with a yellow outline. A representative IBA-1 stain is shown to the right, and the red in the middle denotes the positive pixels determined by the Halo program. **B** Representative images are shown for IBA-1, CD68, GFAP, and AT8 immunostains across control, mild traumatic brain injury (mTBI/mT) and severe traumatic brain injury (sTBI/sT) conditions. **C** The percentage of IBA-1 staining in the orbitofrontal cortex (OFC) grey matter across groups (*p* = 0.0086, *F*(2,18) = 6.274, for repeated measures one-way ANOVA, **p = 0.0024, *t*(18) = 4.534, post hoc test controlling for the false discovery rate). **D** The percentage of CD68 staining across groups (*p* = 0.1458, *F*(1.285,11.57) = 2.391, for repeated measures one-way ANOVA). **E** The percentage of GFAP staining across groups (*p* = 0.1003, *F*(1.456,13.11) = 2.925, for repeated measures one-way ANOVA). **F** The percentage of AT8 staining across groups (*p* = 0.0464, *F*(1.099,9.894) = 5.037, for repeated measures one-way ANOVA, *p = 0.0383, *t*(9) = 2.425, post hoc test controlling for the false discovery rate). **G** Representative image demonstrating a satellite microglia (outlined in red) apposed to a neuron (outlined in orange). **H** Immunofluorescent staining (green = IBA-1 and red = Nissl) and confocal imaging confirmed apposition in three dimensions. **I** The percentage of neurons apposed to a satellite microglia across groups (*p* = 0.0045, *F*(1.690,15.21) = 8.514, for repeated measures one-way ANOVA, *p = 0.0361,* t*(9) = 2.462 and **p = 0.0018, *t*(9) = 4.387, post hoc test controlling for the false discovery rate). Each case is represented with a symbol; solid lines indicate the mean ± SEM. (*n* = 10 control, mTBI and sTBI cases that are age- and sex- matched across groups)

To assess the most prominent pathologies associated with chronic TBI, OFC sections from all cases were stained with IBA-1, CD68, GFAP, and AT8, and the percent area staining in the grey matter was determined and compared across groups (Fig. 1A–B). The OFC was chosen for evaluation given its frequent vulnerability in mild to severe TBI [36, 37]. A significant difference in staining across groups was noted for IBA-1 and AT8 by repeated measures one-way ANOVA testing; post-hoc analysis specifically found a significant increase in the sT compared to the control group (Fig. 1C, F). These differences remained significant only for the IBA-1 staining with removal of cases with CTE from analysis (*p* = 0.0205, one-way ANOVA). Significant differences were not identified for CD68 or GFAP stains (Fig. 1D–E).

When analyzing the IBA-1 images, we noticed not only an increase in staining of microglia, but that many of these microglia appeared to be situated next to neurons (Fig. 1G). These microglia have been denoted as perineuronal satellite microglia in the literature [30, 31], and we turned to 3D immunofluorescent confocal imaging (Fig. 1H) to confirm our impression. Indeed, more neurons were directly in contact with satellite microglia in the TBI groups compared to control, and this relationship was significant with post-hoc testing for both mild and severe TBI with or without CTE cases included (Fig. 1I).

**Fig. 2:**
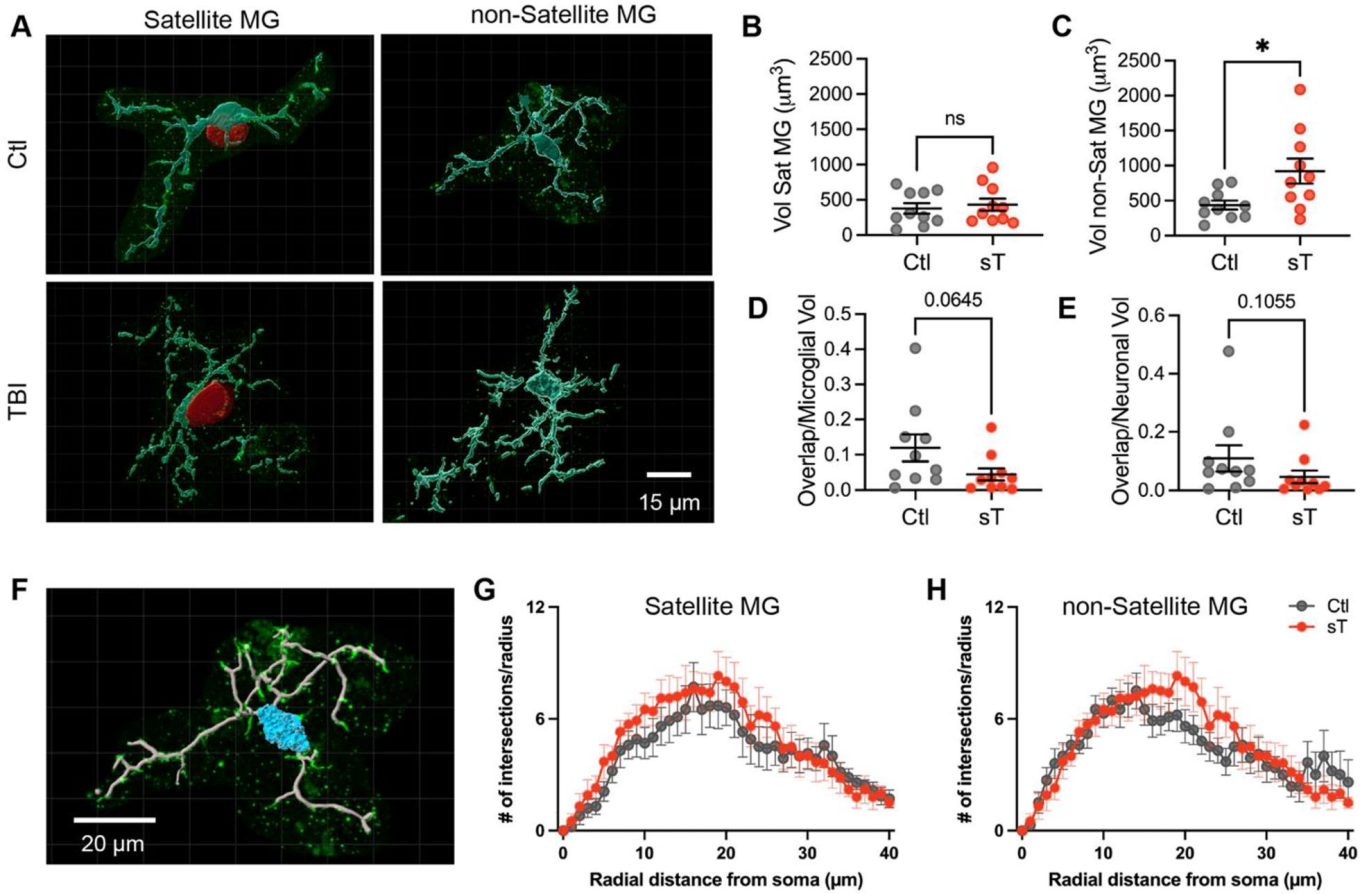
The morphology of satellite and non-satellite microglia in the human orbitofrontal cortex after chronic TBI. **A** Representative Imaris 3D reconstructions of microglia (turquoise) and the apposed neuronal soma (red) for satellite microglia in control and severe TBI cases. These 3D reconstructions were used to calculate microglia, neuronal soma, and overlap volumes. **B** The volume of satellite microglia in control (ctl) and severe TBI (sT) cases (*p* = 0.7015, *t*(9) = 0.3832, paired t-test). **C** The volume of non-satellite microglia in control (ctl) and severe TBI (sT) cases (*p = 0.0181, *t*(9) = 2.883, paired t-test). **D** The ratio of the overlap volume between satellite microglia and the apposing neuronal soma to the microglial volume across groups (*p* = 0.0645, Wilcoxon test). **E** The ratio of the overlap volume between satellite microglia and the apposing neuronal soma to the neuronal soma across groups (*p* = 0.1055, Wilcoxon test). **F** Representative Imaris reconstruction of the soma (blue) and filaments (grey) used to analyze branching in microglia. **G** Scholl analysis plot of satellite microglia in control (black) and severe TBI (red) conditions (*p* = 0.5206, *F*(1,18) = 0.4293, for TBI effect and 0.9832, *F*(40,640) = 0.5782, for distance x TBI effect, mixed-effects analysis). **H** Scholl analysis plot of non-satellite microglia in control (black) and severe TBI (red) conditions (*p* = 0.7120, *F*(1,18) = 0.1406, for TBI effect and 0.2347, *F*(40,640) = 1.160, for distance x TBI effect, mixed-effects analysis). One reconstructed microglia per case is represented with a symbol; solid lines indicate the mean ± SEM in **B**,**C**,**D**,**E**. In **G** and **H**, the mean for each distance is represented by a symbol. (*n* = 10 control and sTBI cases that are age- and sex- matched across groups)

To further understand microglial-neuronal interaction and changes in morphology after TBI, satellite microglia and non-satellite microglia were reconstructed in Imaris (Fig. 2A). Microglia and neuronal soma volumes along with the overlap volume ratios were obtained and compared. As the strongest increase in neurons with satellite microglia was seen in severe TBI, we limited our analysis to control and sT groups. While the individual volumes of non-satellite microglia significantly increased with TBI as expected (Fig. 2C), the volumes of satellite microglia did not change with TBI (Fig. 2B), supporting a differential role for this subtype. The ratio of the volume of overlap to both microglia and neuronal soma volumes showed a weak but insignificant trend of decreasing in the TBI group as compared to the control group, suggesting a reduction in cell–cell interaction after injury (Fig. 2D, E). Scholl analysis was also performed on both satellite and non-satellite microglia to analyze process branching using the filaments tool (Fig. 2F). No differences in branching patterns were found between groups in either satellite or non-satellite microglia (Fig. 2G–H) or in satellite compared to non-satellite microglia (data not shown).

### A mouse model of contusion recapitulates the increase in satellite microglia found in human TBI

**Fig. 3:**
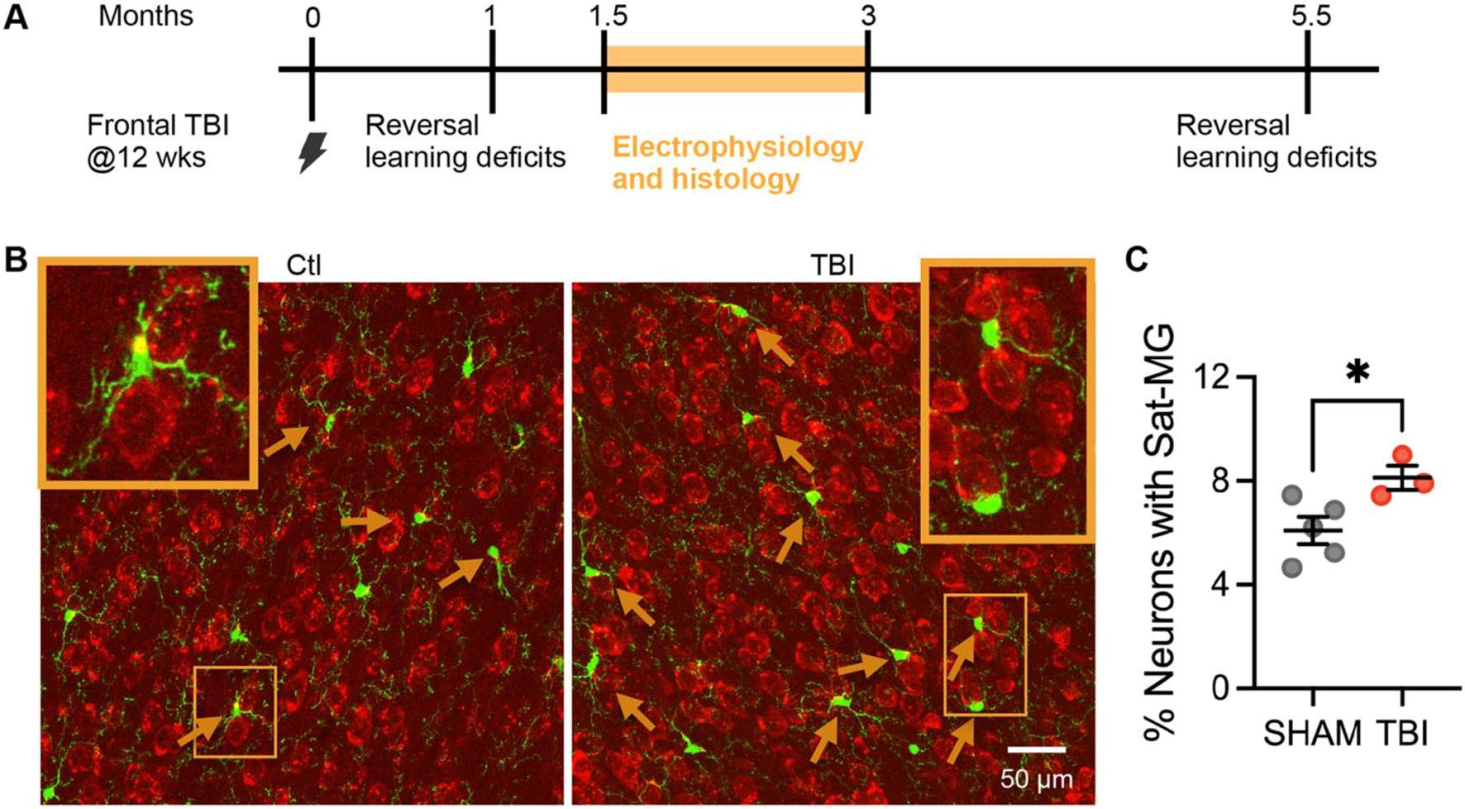
Mouse model recapitulates changes in satellite microglia in the orbitofrontal cortex seen in human TBI. **A** Experimental paradigm and timeline. Adult Tmem-119-GFP mice undergo a sham or frontal controlled cortical impact at ~ 12 weeks of age. In this model, reversal learning deficits (behavior associated with the orbitofrontal cortex (OFC)) have been published at 1 and 5.5 months after injury (1). Thus, all electrophysiology and histology studies were performed between 1.5 and 3 months. **B** Representative immunoflourescent images of the OFC in control and TBI conditions (green = antibody to GFP which is expressed under the Tmem promoter, red = Nissl stain). Orange arrows denote a satellite microglia apposed to a neuronal soma that have been confirmed in 3D. Orange boxes at corners demonstrate an expanded view of this relationship. **C** The percentage of neurons apposed to a satellite microglia across groups (*p = 0.0379, *F*(4,2) = 2.089, unpaired t-test) Each mouse is represented with a symbol and solid lines indicate the mean ± SEM (*n* = 5 sham and 3 TBI animals/group).

To investigate if increased numbers of neurons with satellite microglia after chronic TBI could be reproduced in the OFC in a mouse model, we examined the effect of a frontal controlled cortical impact on transgenic mice expressing GFP under the Tmem119 promoter (expressed only in microglia) [40]. This TBI model has previously been shown to lead to chronic deficits in reversal learning [1], a behavior dependent on the OFC in lesion studies [41]. Adult mice were given sham or frontal controlled cortical impacts at 12–13 weeks of age, and as reversal learning deficits have been identified at one and five and a half months after injury in this model [1], most electrophysiological and histological studies were performed between 1.5 and 3 months (Fig. 3A). A significant increase in the percentage of neurons with satellite microglia was also identified in mice after chronic TBI at this time point (Fig. 3B–C) (as well as at ~ 1 week after injury, see Supplementary Fig. 2).

### Neurons apposed to satellite microglia exhibit reduced excitability but not after TBI

**Fig. 4:**
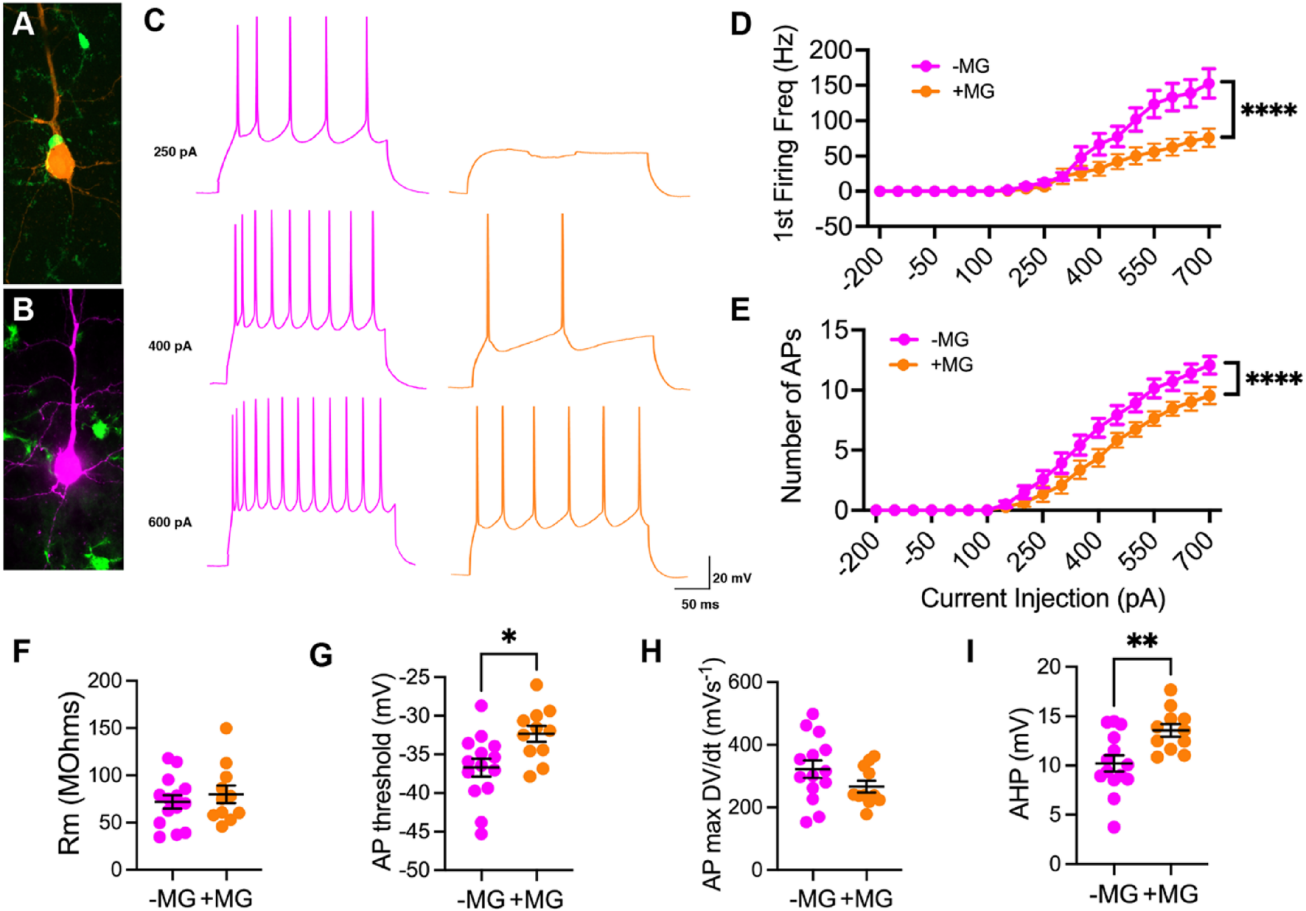
In control conditions, neurons apposed to satellite microglia exhibit reduced excitability. **A** A pyramidal neuron (orange) seen apposed to a satellite microglia (green) is filled with biocytin during recording and this relationship is confirmed with immunoflourescent staining. **B** A pyramidal neuron (pink) away from microglia (green) is filled with biocytin during recording and this relationship is confirmed with immunoflourescent staining. **C** Representative current-clamp responses to depolarizing current steps in neurons away from microglia (pink) and neurons apposed to satellite microglia (orange). **D** The first firing frequency plotted as a function of current injection (*p* = 0.0173, *F*(1,23) = 6.582 for satellite microglia effect, and ****p < 0.0001, *F*(18,414) = 5.897 for interaction between current and satellite microglia, repeated measures 2-way ANOVA). **E** The number of action potentials (AP) plotted as a function of current injection (*p* = 0.0412, *F*(1,23) = 4.680 for satellite microglia effect, and ****p < 0.0001, *F*(18,414) = 3.705 for interaction between current and satellite microglia, repeated measures 2-way ANOVA). **F** The membrane resistance across neurons adjacent to satellite microglia (+ MG) and away from microglia (-MG) (*p* = 0.4923, *F*(13,10) = 1.366, unpaired t-test). **G**–**I** The AP threshold (*p = 0.0117, *F*(13,10) = 1.588, unpaired t-test), maximum rising slope of the action potential (p = 0.1283, *F*(13,10) = 2.829, unpaired t-test) and action potential afterhyperpolarization (AHP) (**p = 0.0054, *F*(13,10) = 2.084, unpaired t-test) calculated from current clamp responses 100 pA above spiking threshold across groups Each neuron is represented with a symbol; solid lines indicate the mean ± SEM. (*n* = 14 neurons away from microglia and 11 neurons apposed to microglia from 13 sham mice).

**Fig. 5:**
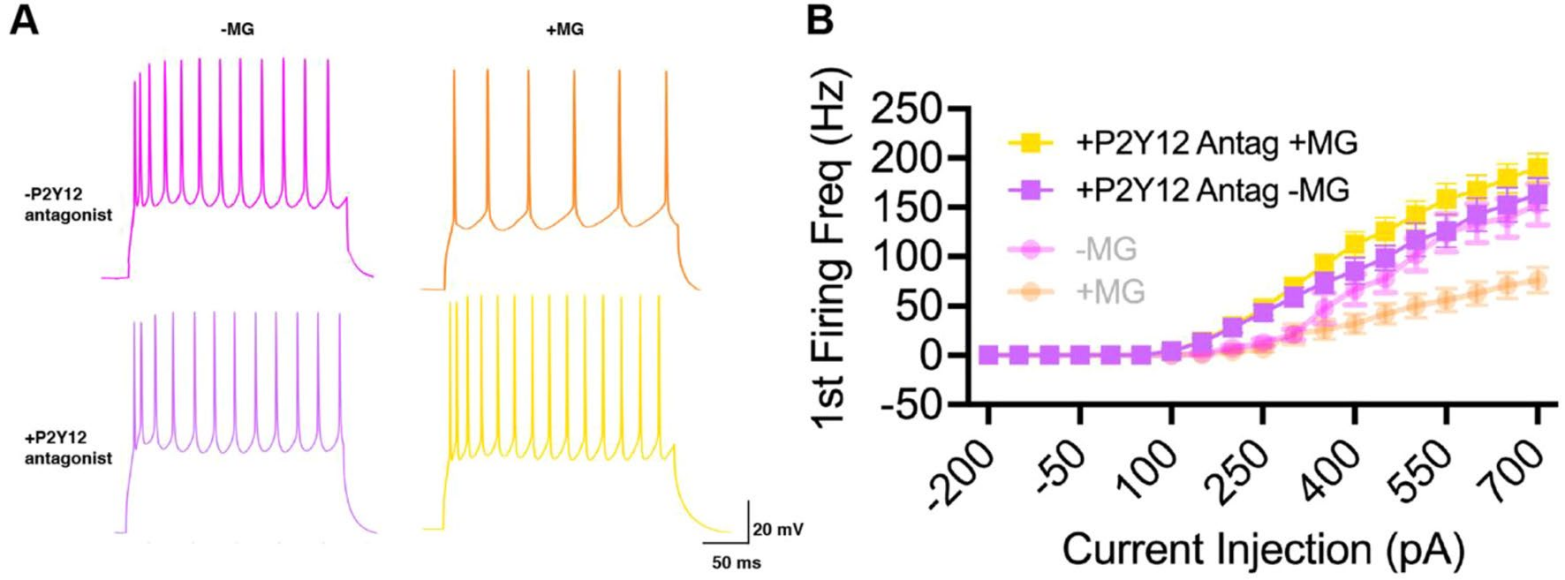
Blocking a microglia-specific receptor abrogates the effect of satellite microglia on neuronal excitability. **A** Representative current-clamp responses to a 600 pA depolarizing current step in neurons away from microglia with (purple) or without (pink) a P2Y12R antagonist- PSB 0739 1 μM added to the aCSF, and neurons apposed to satellite microglia with (yellow) or without (orange) PSB 0739. **B** The first firing frequency plotted as a function of current injection (*p* = 0.4638, *F*(1,21) = 0.5567, for satellite microglia effect, and p = 0.9027, *F*(18,378) = 0.5958, for interaction between current and satellite microglia, repeated measures 2-way ANOVA), data from Fig. 4 are shown with transparency in the background Each neuron is represented with a symbol; solid lines indicate the mean ± SEM. (*n* = 10 neurons away from microglia and 13 neurons apposed to microglia from 7 mice).

After establishing that a mouse model recapitulates the increase in satellite microglia seen in chronic human TBI, we next investigated how these satellite microglia might affect neuronal excitability. Whole cell patch clamp experiments (see Supplementary material for more details on suitability for investigating microglial-neuronal interactions) were performed on in vitro slices of the OFC in sham mice, and using a series of current steps, invoked action potentials of neurons adjacent to and away from satellite microglia were recorded. For both neuron types, as current injection increased, the number of action potentials (APs) increased. However, neurons apposed to satellite microglia showed a significantly lower first firing frequency and significantly fewer action potentials (APs) across current amplitudes as compared to those away from microglia (Fig. 4A–E), a measure of reduced intrinsic excitability [42]. Neurons near microglia also had a significantly increased AP threshold and AP afterhyperpolarization (Fig. 4G, I), while other resting and active membrane properties were not affected (Fig. 4F, H; Supplementary Table 1). This change in excitability is likely a direct effect of satellite microglia, as blocking a microglia-specific receptor, the P2Y12 receptor, lead to no difference in excitability between neurons adjacent to or away from satellite microglia (Fig. 5).

**Fig. 6:**
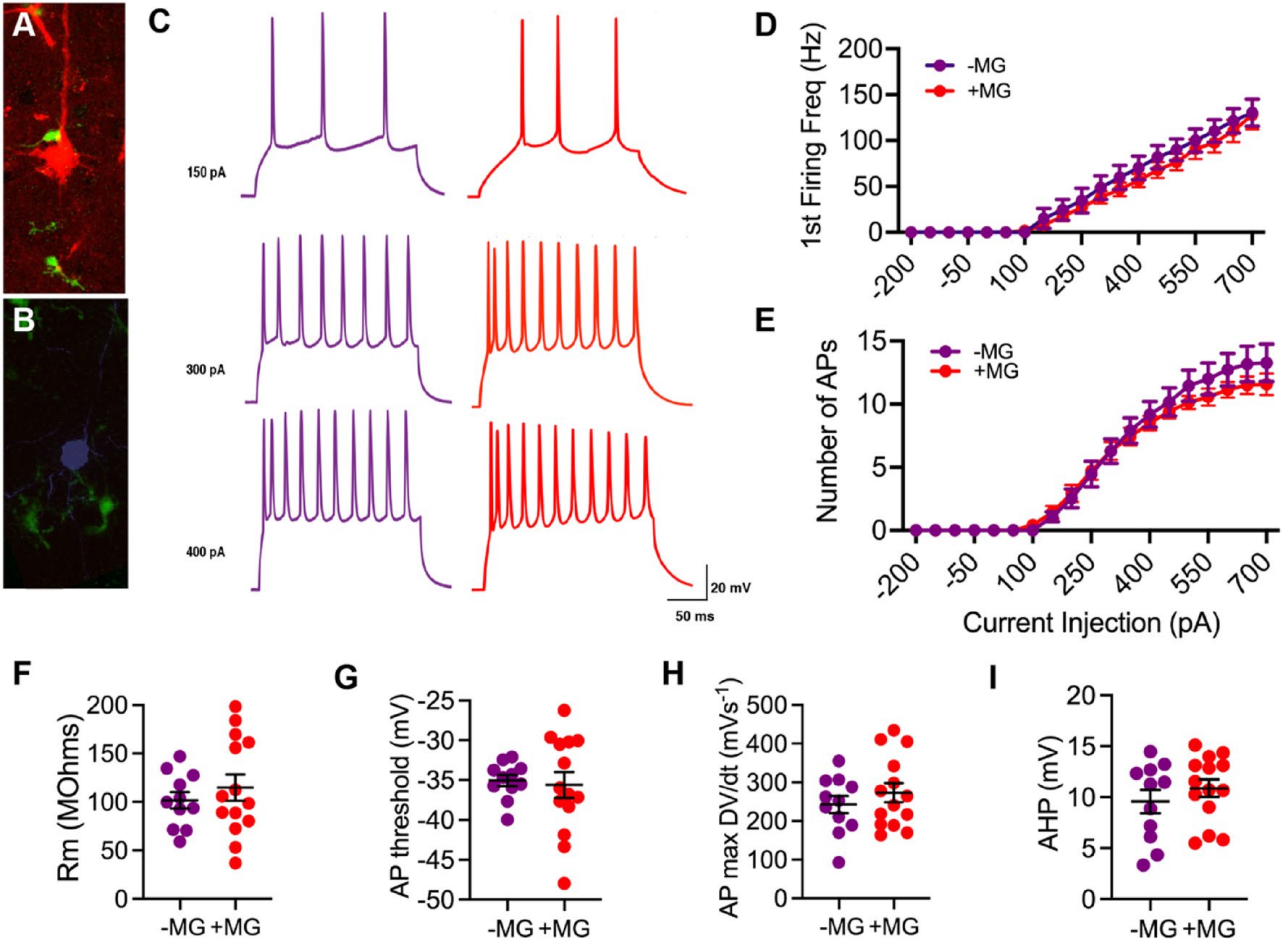
After chronic TBI, neurons apposed to satellite microglia no longer exhibit changes in excitability. **A** A pyramidal neuron (red) seen apposed to a satellite microglia (green) recorded in slices from mice with TBI 2 months prior is filled with biocytin during recording and this relationship is confirmed with immunoflourescent staining. **B** A pyramidal neuron (purple) away from microglia (green) recorded in slices from mice with TBI 2 months prior is filled with biocytin during recording and this relationship is confirmed with immunoflourescent staining. **C** Representative current-clamp responses to depolarizing current steps in neurons away from microglia (purple) and neurons apposed to satellite microglia (red) after chronic TBI. **D** The first firing frequency plotted as a function of current injection (*p* = 0.4516, *F*(1,23) = 0.5864 for satellite microglia effect, and p = 0.9838, *F*(18,414) = 0.4188 for interaction between current and satellite microglia, repeated measures 2-way ANOVA). **E** The number of action potentials (AP) plotted as a function of current injection (*p* = 0.5219, *F*(1,23) = 0.4230, for satellite microglia effect, and *p* = 0.4095, *F*(18,414) = 1.043 for interaction between current and satellite microglia, repeated measures 2-way ANOVA). **F** The membrane resistance across neurons adjacent to satellite microglia (+ MG) and away from microglia (-MG) in chronic TBI (*p* = 0.4469, *F*(13,10) = 3.228, unpaired t-test). **G**–**I** The AP threshold (*p* = 0.7737, *F*(13,10) = 7.060, unpaired t-test), maximum rising slope of the action potential (*p* = 0.3829, *F*(13,10) = 1.569, unpaired t-test) and action potential afterhyperpolarization (AHP) (*p* = 0.3625, *F*(13,10) = 1.411, unpaired t-test) calculated from current clamp responses 100 pA above spiking threshold across groups Each neuron is represented with a symbol; solid lines indicate the mean ± SEM. (*n* = 11 neurons away from microglia and 14 neurons apposed to microglia from 12 TBI mice).

To assess if a similar dampening of excitability occurs after injury, these experiments were repeated in mice with TBI. Current clamp responses to depolarizing steps appeared the same for neurons both adjacent to and away from microglia in the TBI condition, no longer showing a decrease in the first firing frequency or number of APs for neurons near microglia (Fig. 6).

### Neuronal cfos expression mirrors network excitability in both mouse and human tissues

**Fig. 7:**
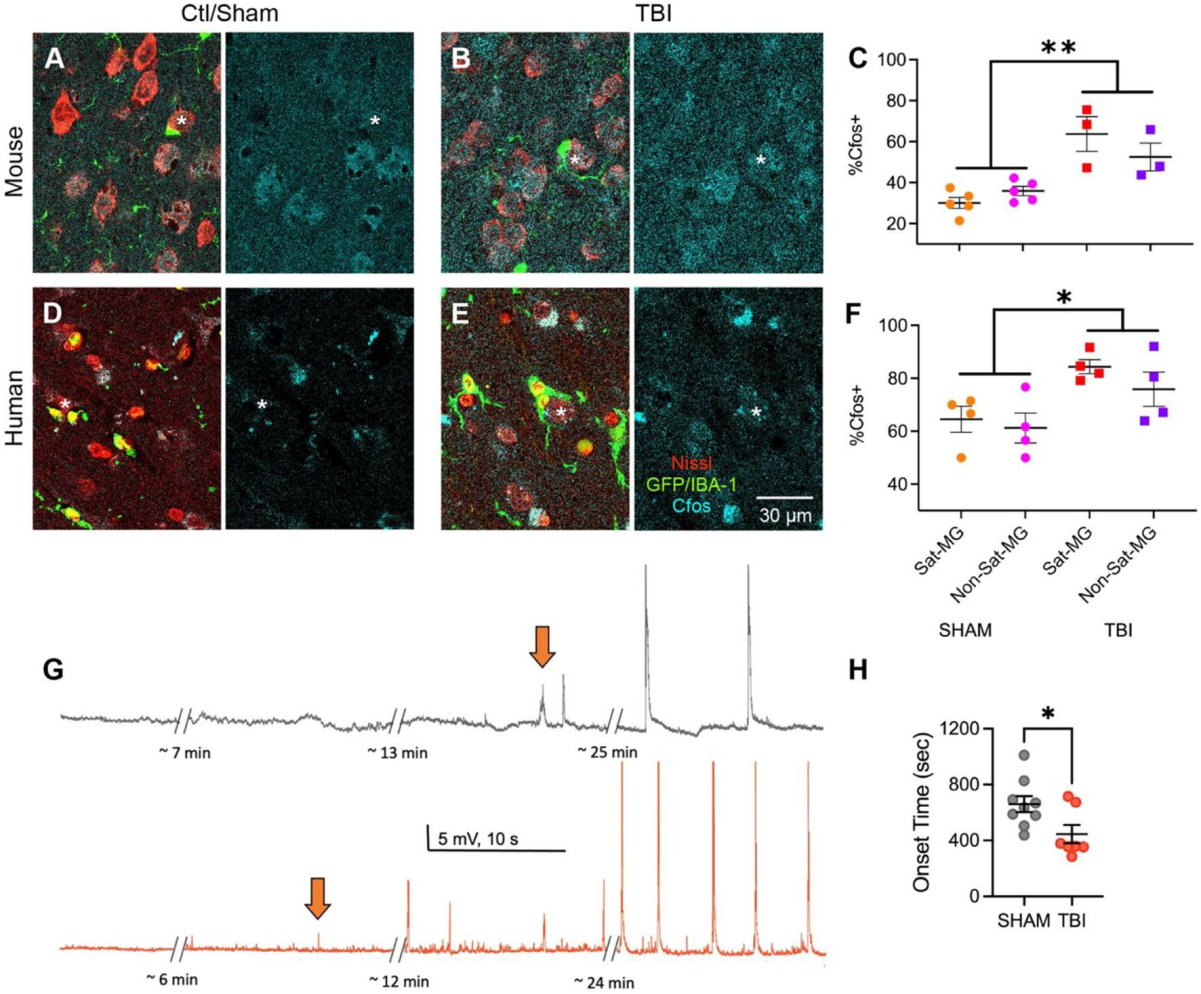
Cfos immunostaining mirrors network excitability. **A** Representative immunostaining for Cfos (cyan), GFP (green), and Nissl (red) in sham and TBI conditions **B** in mice; similar images (IBA-1 instead of GFP) are shown in control and human TBI conditions **D**–**E**; *indicates neurons with satellite microglia. All stains are shown in images on left, while Cfos alone is shown in right image. **C** The percentage of neurons with Cfos positivity are shown for neurons with and without satellite microglia in both sham and TBI conditions in mice (**p = 0.0043, *F*(1,6) = 19.81, TBI effect, 2-way ANOVA (not plotted *p* = 0.0393, *F*(1,6) = 6.896, TBI x MG-association effect)). **F** The percentage of neurons with Cfos positivity are shown for neurons with and without satellite microglia in both control and TBI conditions in human (* *p* = 0.0204, *F*(1,6) = 9.788, TBI effect, 2-way ANOVA). The mean for each subject (counted across multiple images) is represented with a symbol; solid lines indicate the mean ± SEM. (*n* = 5 sham mice, 3 TBI mice, 4 control human tissues, 4 TBI human tissues). **G** Representative current-clamp recordings from layer V pyramidal neurons of the OFC in a sham (gray) and TBI (red) animal showing synchronized input induced with a 0 mM Mg2 + /5 mm K + ACSF starting sooner in the TBI condition (compare timing of orange arrows). **H** The latency from 0 mM Mg2 + /5 mM K + ACSF application until the start of synchronized inputs (*p = 0.0269, *F*(6,8) = 1.004, unpaired t test). Each neuron is represented with a symbol, and solid lines indicate mean ± SEM (*n* = 8 sham and 6 TBI neurons from 3 (sham) and 3 (TBI) animals/group)

To see if changes in excitability measured functionally with whole cell patch clamp could be correlated with immunostaining (a metric that could be performed on our human tissues in lieu of electrophysiology), we analyzed if cfos staining would show differences across sham and TBI conditions in neurons adjacent to and away from satellite microglia. The cfos protein is an immediate early gene that has been correlated with neuronal activity, including activity modulated by the overall presence of microglia [43]. The percentage of cfos-positive neurons was significantly associated with TBI by 2-way ANOVA testing with a higher number of positive neurons in TBI compared to sham for both satellite-associated and not-associated neurons (Fig. 7A–C). This result mirrors the general increased network excitability that occurs after TBI [38], which was replicated here in transgenic Tmem119 mice (Fig. 7G–H). Network excitability in vitro was assessed by measuring the onset time to the development of polysynaptic synchronized events in a random pyramidal neuron in layer V, which occurs gradually over time and increases in frequency when placing the slice in a hyperexcitable solution (0 mM Mg^2+^ and 5 mM K^+^).

However, while there was a significant relationship for TBI with the percentage of cfos + neurons, post-hoc testing was not significant for a statistical difference between satellite-associated neurons compared to neurons without satellite microglia in the sham condition. Thus, cfos staining might be a better indicator of overall activity in the neuron that occurs during network activation, which likely is mediated by synaptic input in addition to the intrinsic excitability measured in Figs.4, 6. Of note, a similar relationship was found in human tissues stained with cfos (we stained the 4 control cases with lowest numbers of satellite microglia-associated neurons and the 4 TBI cases with highest number of satellite microglia-associated neurons), where increased numbers of cfos-positive neurons occur with TBI (Fig. 7D–F), suggesting that the human OFC network also exhibits hyperexcitability as seen in mouse OFC.

### Blocking a microglia-specific receptor also increases network excitability

**Fig. 8:**
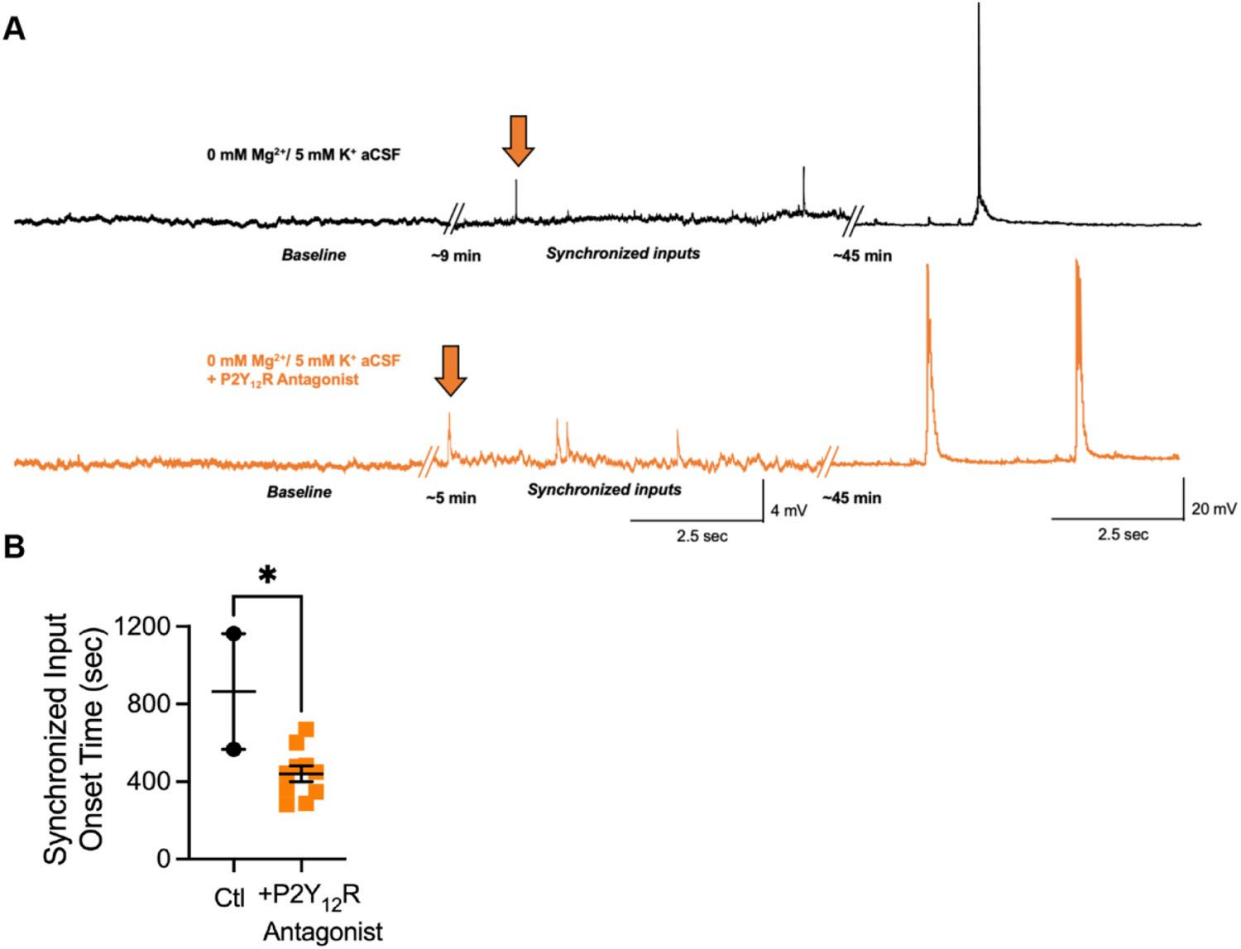
Blocking a microglia-specific receptor increases network excitability. **A** Representative current-clamp recordings from layer V pyramidal neurons of the OFC in control (black) and drug condition (P2Y12R antagonist- PSB 0739, orange) showing synchronized input induced with a 0 mM Mg2 + /5 mm K + ACSF starting sooner in the drug condition (orange arrows). **B** The latency from 0 mM Mg2 + /5 mM K + ACSF application until the start of synchronized inputs (*p = 0.0122, unpaired t test). Each neuron is represented with a symbol, and solid lines indicate mean ± SEM (n = 2 control and 10 drug condition neurons from 6 animals)

To examine if blocking the same microglia-specific receptor that could abrogate the effect of satellite microglia on reducing intrinsic neuronal excitability could affect network function, we assessed the onset time to the development of polysynaptic synchronized events in a hyperexcitable solution (0 mM Mg^2+^ and 5 mM K^+^) while including a P2Y12R antagonist- PSB 0739 in the bath. Compared to a hyperexcitable solution without this antagonist, increased network excitability was observed in vitro as measured by the onset time to polysynaptic synchronized events (Fig. 8).

## Discussion

Here, we evaluated the human neuropathology of chronic TBI, finding a prominent change in microglial response, including an increase in microglial-neuronal interaction characterized by more neurons with satellite microglia. This increased interaction was even found in mild TBI cases and could be reproduced in a mouse model of frontal lobe contusion. Further mechanistic investigation in this mouse model demonstrated that satellite microglia association reduced the intrinsic excitability of neurons, but this effect was lost after chronic TBI. Similarly, network excitability measured by electrophysiology in mice, as well as cfos expression in both mouse and human tissues was increased after chronic TBI, supporting a possible role for this loss of homeostatic control by satellite microglia in the maladaptive circuit changes that occur after TBI.

Chronic changes in microglia have often been identified after moderate/severe or repetitive human TBI as measured by expression of CR3/43- and/or CD68 and other metrics [5, 44, 45]. However, “mild” human TBI has not been fully evaluated nor the specific changes in microglial-neuronal interaction after injury found here with the increased presence of satellite microglia. Our data also continue to add to the literature supporting a unique role for satellite microglia [30, 33] given their differential response in volume change after TBI: while non-satellite microglia increase their volume consistent with a “reactive” state, satellite microglia do not.

Increased satellite microglia have been found in other disease states, including acute neuroinflammation induced by systemic lipopolysaccharide (LPS) administration, a model of complex febrile seizures and a murine model of encephalitis [29, 46, 47]. In the context of LPS and febrile seizures, the increase in satellite microglia is transient and is thought to help restore function. Specifically, injury induced after LPS administration is associated with reduced cortical neuron apoptosis after a second injury event, and inhibitory synapse stripping by satellite microglia may increase neuronal viability signaling [46]. However, in the encephalitis model, this relationship is prolonged and satellite microglia and phagocytes continue to enwrap infected neurons in the deep cerebellar nuclei for several weeks [47]. In this model, pharmacologically reducing microglia/phagocyte activation and associated numbers of satellite cells improved motor performance, suggesting that while neuronal viability may be improved, detrimental effects on the function of neuronal circuits might ensue [47].

Direct assessment of electrical activity in neurons adjacent to satellite microglia has been limited. Wan et al*.* used whole-cell patch clamp to determine functional changes in synaptic inhibition [29]. In control conditions, neuronal relationship with satellite microglia did not change the frequency of spontaneous or miniature inhibitory postsynaptic currents (IPSCs), but after inducing febrile seizures, satellite microglia association reduced the frequency of both s-IPSC and m-IPSCs. They also assessed action potential production by analyzing the frequency as a function of current injection, similar to our investigation here. In contrast to our findings, they recorded no difference between the excitability of neurons associated with and those without satellite microglia in the control group but did show a decrease in excitability in neurons with satellite microglia in overexcited circuits with febrile seizures that appeared to be due to blocking the depolarizing effect of tonic GABA currents at the very young age of their model (postnatal day P8). The differences in our data are likely related to development as we are studying adult mice known to not only have fully-developed GABA synapses with inhibitory reversal potentials [48], but also have very different action potential waveforms and underlying ion channels contributing [49]. More recently, this same research group used in vivo calcium imaging in young adult mice to examine activity in neurons adjacent to and away from satellite microglia finding a higher frequency of calcium spikes in neurons apposed to satellite microglia compared to those away, which the authors suggest is likely secondary to a reduction in GABAergic input that accompanies satellite microglia association. Certainly, much more work needs to be done to elucidate the role of both intrinsic changes in action potentials along with the interaction with synaptic excitation/inhibition to understand the complex role of satellite microglia in regulating circuit activity both in physiologic conditions and after injury/disease. While we have not assessed the effect of satellite microglia on inhibition, we have clearly found a change in intrinsic excitability regulation that is lost after chronic TBI and is associated with the presence of a hyperexcitable network.

Exactly how satellite microglia modulate neuronal action potential production is unclear. Given our observation that direct antagonism of a microglial-specific receptor abrogated the differential response in neurons with and without satellite microglia, we favor that satellite microglia directly contribute to modifying neuronal excitability rather than preferentially associating with neurons that already have lower excitability. The action potential threshold and afterhyperpolarization were both increased in neurons adjacent to satellite microglia, suggesting a change in a voltage- or calcium-dependent ion channel, rather than only an effect on stripping inhibitory synapses and modifying tonic inhibition as proposed in the prior studies. However, tonic inhibition can exhibit outward rectification and effect the gain of action potential response in complex ways [50, 51], so this possibility will need to be investigated in future studies. A substantial proportion of these satellite microglia are also known to have processes that wrap around the axon initial segment (AIS) [32], the region of action potential production, raising the possibility that AIS-associated ion channels are modified with satellite microglia interaction. Finally, microglia in general have been found to work through the purinergic system, ultimately activating adenosine receptors and possibly G-protein-gated inwardly rectifying potassium (GIRK) channels to reduce cultured neuronal activity and in vivo seizure production [27]. If this pathway is amplified specifically in satellite microglia will also require consideration.

Why satellite microglia lose the ability to regulate excitably after injury is also uncertain. We found a nonsignificant trend towards a reduction in possible cell–cell interaction after TBI (Fig. 2D–E). Thus, perhaps there is less direct membrane interaction that contains the protein mediators needed to trigger downstream changes in excitability. If the purinergic system is involved, reduction in homeostatic proteins, including the P2Y12 receptor, can occur after TBI and these receptors have been implicated in somatic neuronal interaction and control of neuronal excitability [43, 52–54]. Certainly, our data blocking the P2Y12 receptor phenocopied the same results seen in chronic TBI, supporting further investigation. Spatial transcriptomic approaches will be invaluable for deeply phenotyping the interactions of satellite microglia with neurons to address their change in behavior after TBI and further interrogate how these microglia or similar or different from non-satellite microglia.

Our study has limitations that should be addressed. Only male human brain donors were available to age-match across injury severity for analysis, and future studies will be needed to see if the same conclusions can be drawn in the female brain after injury. In addition, whether the effects seen here are truly due to soma-soma satellite microglial-neuronal association or whether these neurons just happen to have more somatic contact with microglial processes leading to this effect is a question that will need to be addressed with future research.

## Conclusions

In conclusion, we have established that changes in microglia including increased microglial-neuronal interaction in the form of satellite microglia are a hallmark of chronic traumatic brain injury, even mild injury. These satellite microglia play a role in neuronal excitability and this likely homeostatic function is lost after injury. The mechanisms underlying this neuronal regulation and change after injury need to be fully investigated to inform targeted therapies for chronic circuit dysfunction and progressive decline after TBI.

## Supplementary Information

Below is the link to the electronic supplementary material.

Former article version (PDF 4904 KB)

## Data Availability

The datasets used and/or analysed during the current study are available from the corresponding author on reasonable request.

## Abbreviations

TBI: Traumatic brain injury
NDD: Neurodegenerative disease
Sat-MG: Satellite microglia
UW: University of Washington
OFC: Orbitofrontal cortex
CTE: Chronic traumatic encephalopathy
aCSF: Artificial cerebral spinal fluid
AP: Action potential
AHP: Afterhyperpolarization
