# Supplementary material for: Satellite microglia: marker of traumatic brain injury and regulator of neuronal excitability
Source: J Neuroinflammation. 2025 Jan 16;22:9. doi: 10.1186/s12974-024-03328-9 (PMC11740464; doi:10.1186/s12974-024-03328-9)
Supplement: Supplementary file 1 — Former article version (PDF 4904 KB) [file 12974_2024_3328_MOESM1_ESM.pdf]

Supplementary Material

In vitro slices to examine satellite microglial-neuronal interactions

In vitro slices are the gold standard for performing whole cell patch clamp to assess intrinsic neuronal excitability. The preparation of *in vitro* slices requires slicing of the brain into thin sections, raising questions of whether the slice procedure by itself may affect microglial phenotypes that could obscure the chronic *in situ* response of microglia to TBI that we are really interested in studying. To ensure feasibility, 250 micron slices were prepared for the *in vitro* slice preparation from control Tmem-119EGFP mice. The slices were held for 2, 3, 4, or 5 hrs in oxygenated aCSF and placed in 4% paraformaldehye (PFA) for 24-48 hours before being rinsed and stained. Images from these slices were compared to images taken from cryosections obtained from mice that underwent cardiac perfusion and the same staining procedure. Similar to others,[1] there was no evidence of substantial microglial activation from the *in vitro* preparation alone up to 5 hours after slicing, specifically including any changes in the percentage of neurons interacting with satellite microglia, the percentage of satellite microglia of total microglia, or the area of interaction between satellite microglia and neurons (Supplemental Figure 1), supporting use of this tool to investigate satellite microglia effect on neuronal function.

References

1. Rotterman TM, Alvarez FJ. Microglia Dynamics and Interactions with Motoneurons Axotomized After Nerve Injuries Revealed By Two-Photon Imaging. *Sci Rep*. May 26 2020;10(1):8648. doi:10.1038/s41598-020-65363-9

Supplementary Tables and Figures

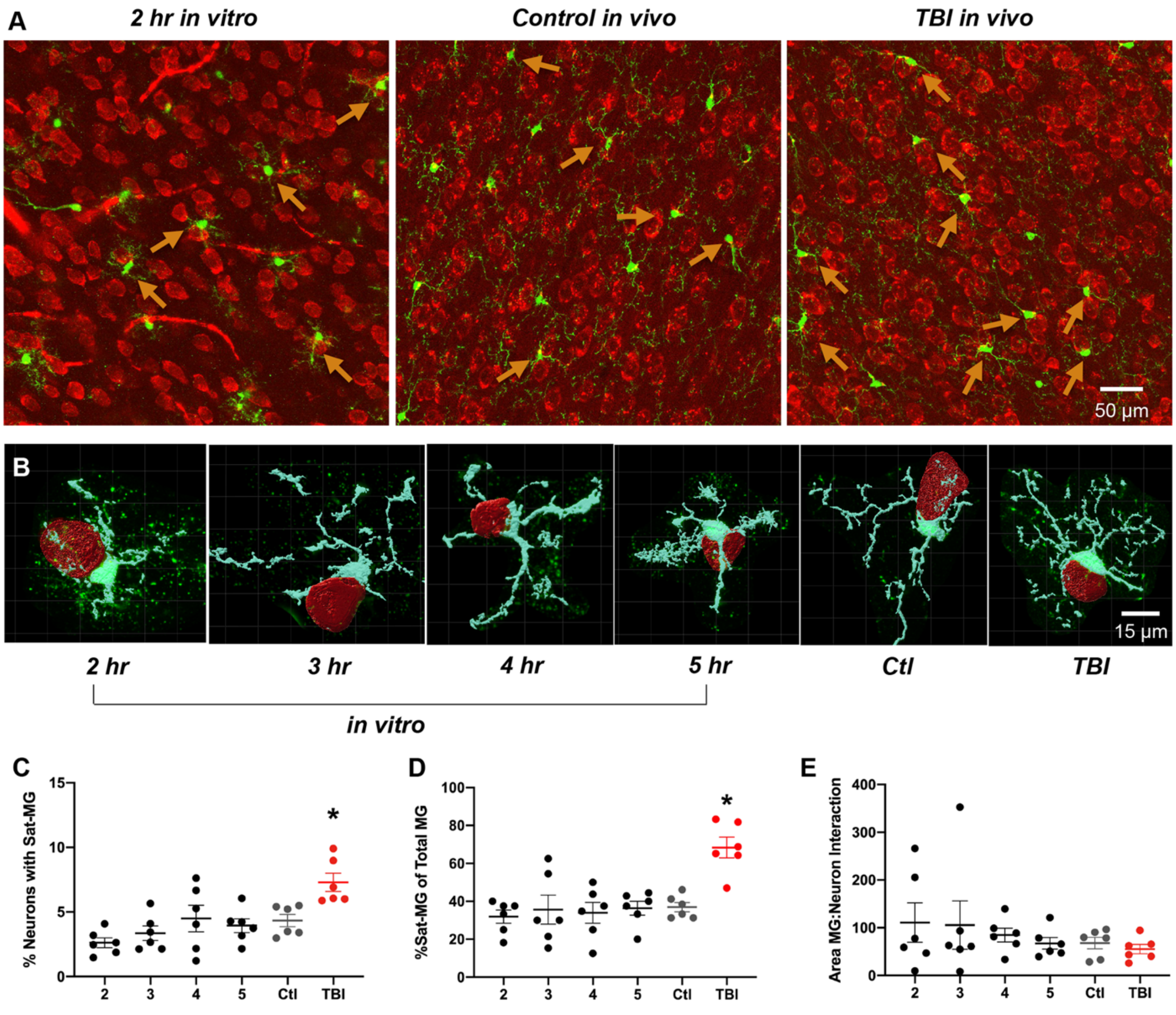

Supplementary Figure 1 *In vitro* preparation does not alter satellite microglia. A) Representative images of microglia (green-GFP in Tmem119-EGP mice) and neurons (red, Nissl) in a slice preparation prepared for electrophysiological recordings and fixed 2 hrs after preparation, and a control and TBI mouse perfused for histology. B) Imaris reconstructions of the surface area of the satellite microglia and adjacent neuronal soma. C) % Neurons with satellite microglia, and D) % microglia that are satellite are not altered by the *in vitro* preparation across 2-5 hours before fixation and similar to control, while the TBI condition is significantly different (* $p = 0.0005$, $F(5,30) = 1.595$ and $p = 0.0001$, $F(5,30) = 0.8721$ respectively, one-way ANOVA). E) The surface area of interaction was also not altered by the *in vitro slice* preparation ($p = 0.6866$, $F(5,30) = 1.043$, one-way ANOVA).

Supplementary Table 1 Intrinsic properties of neurons apposed and away from satellite microglia in control and TBI conditions. Values are mean (SEM) or *median (95% CI). Statistical test is indicated with associated p-value.

	-MG Sham	+MG Sham	*p-value*	F/U statistic	-MG TBI	+MG TBI	*p-value*	F/U statistic
**Action potential properties**								
half width (msec)	0.79 (0.05)	0.88 (0.05)	*0.2650 (*unpaired t-test)	*F*(13,10)= 0.7938	1.22 (0.18)	1.01 (0.07)	*0.2850* (Welch's t-test)	*F*(13,10)= 4.947
amplitude (mV)	72.63 (2.43)	67.50 (1.90)	*0.1250 (*unpaired t-test)	*F*(13,10)= 0.2520	69.46 (1.94)	69.57 (2.48)	*0.9733* (unpaired t-test)	*F*(13,10)= 2.066
threshold (mV)	-36.71 (1.16)	-32.33 (1.03)	***0.0117*** *(*unpaired t-test)	*F*(13,10)= 1.588	-35.04 (0.69)	-35.61 (1.63)	*0.7530* (Welch's t-test)	*F*(13,10)= 7.060
rising slope (mV/s)	322.2 (27.67)	266.4 (18.56)	*0.1283 (*unpaired t-test)	*F*(13,10)= 2.829	242.6 (22.31)	273.1 (24.77)	*0.3820* (unpaired t-test)	*F*(13,10)= 1.569
falling slope (mV/s)	-97.82 (9.17)	-78.49 (5.48)	*0.1053 (*unpaired t-test)	*F*(13,10)= 3.569	-67.58 (8.94)	-74.90 (8.05)	*0.5494* (unpaired t-test)	*F*(13,10)= 1.031
spike AHP (mV)	10.21 (0.83)	13.57 (0.65)	***0.0054*** (unpaired t-test)	*F*(13,10)= 2.084	9.58 (1.15)	10.89 (0.86)	*0.3625* (unpaired t-test)	*F*(13,10)= 1.411
adaptation index	*1.34 (1.26-1.90)	*1.25 (1.11-1.62)	*0.2022 (*Mann-Whitney)	*U* = 53	*1.31 (1.17-2.99)	*1.44 (1.19-1.66)	*0.9786 (*Mann-Whitney)	*U* = 76
**Passive properties**								
resting potential (mV)	-66.08 (1.81)	-65.23 (1.64)	*0.7390* (unpaired t-test)	*F*(13,10)= 1.552	-69.27 (1.52)	-64.76 (1.63)	*0.0604* (unpaired t-test)	*F*(13,10)= 1.477
membrane resistance (MΩ)	71.88 (7.06)	79.87 (9.31)	*0.4923* (unpaired t-test)	*F*(13,10)= 1.366	101.62 (8.48)	114.81 (13.51)	*0.4469* (unpaired t-test)	*F*(13,10)= 3.228
tau (msec)	10.43 (1.42)	10.52 (1.42)	*0.9621* (unpaired t-test)	*F*(13,10)= 1.259	11.82 (1.13)	13.10 (1.82)	*0.5816* (unpaired t-test)	*F*(13,10)= 3.284

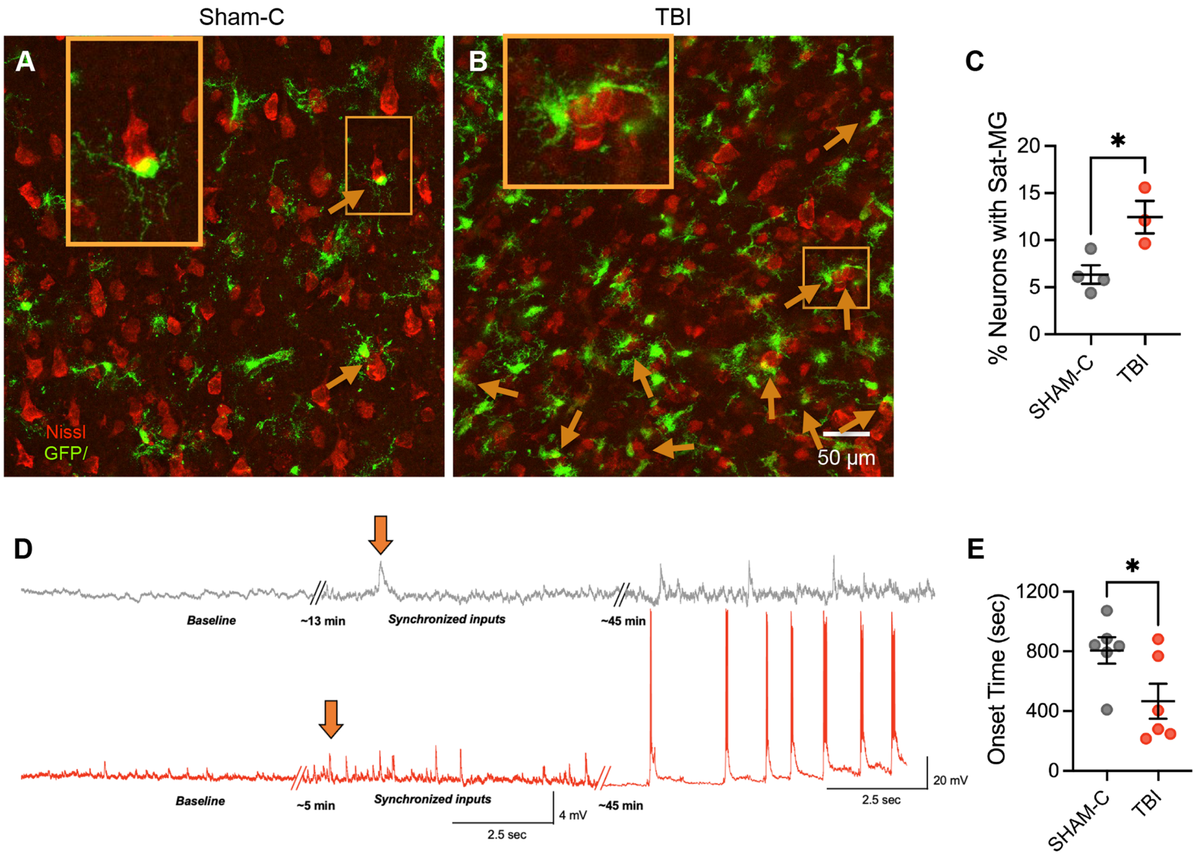

Supplementary Figure 2 Increased satellite microglial interactions and network excitability are observed at 1 week after TBI compared to a sham that underwent full craniectomy. A-B) Representative images of microglia (green-GFP in Tmem119-EGP mice) and neurons (red, Nissl) in slice preparations from control and TBI mice, 1 week after surgery. C) % Neurons with satellite microglia are increased 1 week after TBI(* $p = 0.0219$, $F(2,3) = 2.293$, unpaired t-test). D) Representative current-clamp recordings from layer V pyramidal neurons of the OFC in a sham (gray) and TBI (red) animal showing synchronized input induced with a 0 mM $Mg2+/5$ mm K+ ACSF starting sooner in the TBI condition (orange arrows). E) The latency from 0 mM $Mg2+/5$ mM K+ ACSF application until the start of synchronized inputs (* $p = 0.0436$, $F(5,5)= 1.753$, unpaired t test). Each slice is represented with a symbol and solid lines indicate the mean ± SEM (n=3-6 per condition from 2 mice per group).
